# Elevated stress hyperglycemia ratio predicts intensive care unit admission after surgery for gastrointestinal tumors: an INSPIRE database analysis

**DOI:** 10.3389/fmed.2026.1794194

**Published:** 2026-03-16

**Authors:** Jing Lin, Junfan Chen, Weide Lin, Bixia Lin

**Affiliations:** 1 Department of Anesthesiology, The First Hospital of Putian City, Putian, China; 2 Department of Medical Equipment, The First Hospital of Putian City, Putian, China; 3 Department of Ultrasonography, The First Hospital of Putian City, Putian, China

**Keywords:** admission risk, cohort studies, gastrointestinal tumors, intensive care unit, postoperative, prediction, preoperative, stress hyperglycemia ratio

## Abstract

**Introduction:**

Gastrointestinal tumors are a major global health burden, with surgery as the main curative treatment. The Stress Hyperglycemia Ratio (SHR) has prognostic value in critical illnesses, but its association with postoperative intensive care unit (ICU) admission in gastrointestinal tumor patients remains unclear. This study investigates the relationship between preoperative SHR and ICU transfer risk after gastrointestinal cancer surgery.

**Methods:**

This retrospective cohort study analyzed data from 2,102 gastrointestinal tumor surgery patients in the INSPIRE database. Multivariable logistic regression examined the association between SHR and ICU admission, with models progressively adjusted for confounders. A generalized additive model explored potential nonlinearity. The model’s predictive performance was assessed using receiver operating characteristic (ROC) curves and calibration plots with bootstrapping. Subgroup and sensitivity analyses were performed to evaluate the consistency of the findings.

**Results:**

In fully adjusted models, elevated preoperative SHR was independently associated with increased ICU admission risk. Each 0.1-unit increase in SHR corresponded to an odds ratio (OR) of 1.08 (95% CI: 1.04–1.13, *p* < 0.001). Compared to the lowest tertile, patients in the highest SHR tertile (≥1.04) had an OR of 1.43 (95% CI: 1.04–1.97, *p* = 0.03). The dose–response relationship was linear (P for nonlinearity >0.05). The prediction model incorporating SHR demonstrated good discrimination with an AUC of 0.895 and good calibration with a mean absolute error of 0.009. Subgroup analysis indicated significant interaction effects for age and sex.

**Conclusion:**

Preoperative SHR is a strong and independent predictor of postoperative ICU admission in patients undergoing surgery for gastrointestinal tumors, exhibiting a linear dose–response relationship. The multivariable model incorporating SHR showed excellent predictive performance, with an AUC of 0.895 and good calibration. Integrating SHR assessment into preoperative evaluation could enhance risk stratification, guide perioperative management, and optimize critical care resource allocation for this patient population.

## 1 Introduction

Gastrointestinal tumors represent a major global health burden, accounting for approximately 26% of global cancer incidence and 35% of cancer-related deaths annually (1, 2). Current epidemiological trends reveal a rising incidence among young individuals (≤40 years), where cases are often sporadic and associated with poor dietary habits and low socioeconomic status (1–3). Furthermore, marked geographic and sex-based disparities exist in this disease. East Asia bears the heaviest burden, contributing 48.4% of global gastrointestinal cancer cases, with particularly elevated rates of stomach, liver, esophageal, and gallbladder cancers (4). Regarding sex differences, the burden is significantly higher in men, with a male-to-female incidence ratio reaching 6.7:1 for esophageal adenocarcinoma, and a similar trend observed for colorectal cancer incidence (5). Treatment strategies vary by tumor type, stage, and molecular profile, ranging from endoscopic submucosal dissection (ESD) for early-stage tumors to multimodal approaches for neuroendocrine neoplasms (6, 7). Regardless of the therapeutic strategy employed, postoperative complications remain pivotal determinants of recovery and length of hospital stay. The need for postoperative transfer to the intensive care unit (ICU), often prompted by such complications, is a strong indicator of poor prognosis, with direct ICU admission rates following tumor resection reaching as high as 40%, underscoring the magnitude of this clinical challenge. These patients generally have poor prognoses, particularly within the emergency surgery subgroup, where mortality rates reach 32.5% for emergency resections and exceed 40% for emergency palliative procedures (8). ICU admission not only reflects severe physiological derangement but also significantly increases healthcare costs and prolongs hospital stays. Stress-induced hyperglycemia, a common metabolic disturbance in the perioperative period, has been demonstrated to be closely associated with an increased risk of postoperative complications such as infections and cardiovascular events, establishing it as a key pathophysiological mechanism contributing to the aforementioned adverse outcomes (9–11). Consequently, the accurate identification of patients at risk for stress-induced hyperglycemia and the timely implementation of preventive interventions are critical for enhancing clinical decision-making, improving patient prognosis, and optimizing the allocation of healthcare resources.

Among the various physiological disturbances contributing to this outcome, stress hyperglycemia plays a significant role. Stress hyperglycemia is a common physiological response to critical illness or acute stress. Traditionally assessed based on an absolute elevation in admission or fasting blood glucose, this approach is inherently confounded by the patient’s underlying glycemic status and thus fails to accurately represent the severity of acute stress (12). The underlying pathophysiology linking stress hyperglycemia to adverse outcomes is multifactorial. The surge of counter-regulatory hormones (such as cortisol and glucagon) and pro-inflammatory cytokines during surgical stress induces insulin resistance and promotes hepatic gluconeogenesis (13, 14). This hyperglycemic environment can exacerbate oxidative stress, endothelial dysfunction, and platelet aggregation, while also impairing immune function by compromising neutrophil and macrophage activity (1). These mechanisms collectively predispose patients to an increased risk of cardiovascular instability, infections, and multi-organ dysfunction, all of which are common triggers for postoperative ICU admission (14).

The recently proposed stress hyperglycemia ratio (SHR) is a novel biomarker designed to quantify relative hyperglycemia by relating acute blood glucose levels to long-term glycemic control as measured by glycated hemoglobin (HbA1c). By accounting for an individual’s chronic glucose background, SHR generally demonstrates superior prognostic performance compared to conventional metrics such as fasting glucose (12, 15). For instance, in patients with ischemic stroke, SHR remained independently associated with clinical outcomes after multivariable adjustment, whereas absolute blood glucose levels did not (15). Similarly, in acute intracerebral hemorrhage, SHR has been correlated with both in-hospital mortality and hematoma expansion (16). The predictive value of SHR has also been extensively validated in surgical populations. Xia et al. (17) analyzed 356 patients admitted to the ICU after esophagectomy for esophageal squamous cell carcinoma and found that patients in the high SHR group (≥ 1.14) had significantly higher 30-day and 90-day mortality rates compared to the low SHR group (10.5% vs. 1.3, 20.0% vs. 5.8%, both *p* < 0.001); multivariable analysis showed that each 0.1-unit increase in SHR was associated with a 4.4% increase in 30-day mortality risk (OR = 1.044, 95% CI: 1.036–1.069). Roberts et al. (18) further quantified the degree of stress-induced hyperglycemia associated with different surgical categories, revealing significantly elevated SHR in patients undergoing colectomy (SHR = 1.62 ± 0.48) and cardiac surgeries (coronary artery bypass grafting 1.56 ± 0.43, aortic valve replacement 1.71 ± 0.33, mitral valve replacement 1.75 ± 0.34), with the degree of stress hyperglycemia being approximately 40% greater than that observed in medical cohorts. Additionally, Stretton et al. (19) analyzed 52, 145 surgical patients and demonstrated that in non-diabetic patients, hyperglycemia was associated with an increased risk of ICU admission (OR = 1.95, 95% CI: 1.40–2.72), highlighting the need for relative measures such as SHR. This accumulating evidence indicates that SHR more effectively captures the prognostic influence of stress-induced hyperglycemia across diverse clinical settings, including critical care, cardiovascular, and neurological diseases (9, 20). Despite these established associations, the specific role of SHR in predicting postoperative ICU admission among patients undergoing surgery for gastrointestinal tumors remains unexplored. Therefore, this study aims to investigate the association between preoperative SHR levels and ICU transfer in this surgical population, with the goal of providing a quantitative basis for developing precise risk stratification and individualized perioperative management strategies.

## 2 Materials and methods

### 2.1 Study design and population

This retrospective cohort study utilized data from the publicly available Integrated Perioperative Surgical Patient Information Registry (INSPIRE) database. This high-quality clinical repository from Seoul National University Hospital, South Korea, encompasses comprehensive perioperative data for approximately 130,000 surgical cases between January 2011 and December 2020, including demographic profiles, vital signs, laboratory results, and medication records, facilitating the investigation of risk factors for perioperative outcomes (21). During database construction, based on the decision of the Institutional Data Review Board, 50% of the original patient population was randomly excluded to constitute the publicly released dataset. Building on this, the present study further included 9,439 patients aged 18–90 years who underwent gastrointestinal tumor surgery (based on ICD-10-CM codes: C15–C21 and D00). Subsequently, patients with missing blood glucose data (*n* = 625) and missing HbA1c data (*n* = 6,712) were excluded. A total of 2,102 patients were ultimately included in the final analytical cohort. The study protocol was reviewed and approved by the Institutional Review Board of Seoul National University Hospital (IRB No. H-2210-078-1368). Due to the retrospective nature of the study design, the requirement for written informed consent was waived. Furthermore, as the study involved the analysis of publicly available, de-identified data, no additional approval from a medical ethics committee was required. This manuscript was prepared in accordance with the Strengthening the Reporting of Observational Studies in Epidemiology (STROBE) guidelines (22).

### 2.2 Covariates and outcome measures

Data were extracted from the INSPIRE database using Structured Query Language (SQL), with a data extraction window from January 2011 to December 2020. All laboratory parameters were the first measurements obtained after hospital admission and prior to surgery. The extracted covariates included: age (years), sex, body mass index (BMI, kg/m^2^), American Society of Anesthesiologists (ASA) physical status classification, use of general anesthesia, operation time (minutes), emergency operation, intraoperative use of vasoactive drugs, heart rate (HR, bpm), systolic blood pressure (SBP, mmHg), respiratory rate (RR, bpm), oxygen saturation measured by pulse oximetry (SpO₂, %), hemoglobin (Hb, g/dL), white blood cell count (WBC, ×10^9/L), total protein (TP, g/dL), total bilirubin (TBIL, μmol/L), serum creatinine (Scr, mg/dL), serum potassium (K, mmol/L), serum sodium (Na, mmol/L), serum calcium (Ca, mmol/L), activated partial thromboplastin time (APTT, s), international normalized ratio (INR, s), and histories of hypertension, diabetes mellitus (DM), cardiovascular disease (CVD), chronic obstructive pulmonary disease (COPD), chronic kidney disease (CKD), estimated blood loss (EBL), and infusion volume. The SHR was calculated using the following formula: SHR = Admission Blood Glucose (ABG, mmol/L)/[1.59 × Glycated Hemoglobin (HbA1c, %) − 2.59] (23). The primary outcome of this study was postoperative admission to the ICU. Postoperative ICU admission was defined as the first transfer to the ICU at any time point from the end of surgery until hospital discharge.

### 2.3 Statistical analysis

The normality of continuous variables was assessed using the Shapiro–Wilk test. Normally distributed variables are presented as mean ± standard deviation and were compared between groups using the Student’s *t*-test or one-way analysis of variance (ANOVA). Non-normally distributed variables are presented as median (interquartile range, IQR) and were compared using the Kruskal-Wallis H test. Categorical variables are presented as frequencies and percentages, and group comparisons were performed using the chi-square test or Fisher’s exact test, as appropriate. Prior to modeling, multicollinearity was assessed, which confirmed the absence of severe multicollinearity, as all variance inflation factor (VIF) values were below 5 (Supplementary Table S1). Additionally, there was significant insufficiency in some covariates: the missing rate of blood loss was 21.9%, and the missing rates of other covariates ranged from 0.5 to 5.61%. To address this issue, we employed multiple imputations for the missing covariates. Specifically, we generated five sets of imputed values for missing data. One of the imputed datasets was then used for the subsequent statistical analysis.

The association between SHR and postoperative ICU admission was first evaluated using univariable logistic regression. Subsequently, a series of multivariable logistic regression models were constructed to progressively adjust for potential confounders. Model I adjusted for covariates whose inclusion altered the effect estimate of SHR by >10%. Model II further adjusted for all covariates with a *p* value <0.1 in the univariable analysis. Model III represented the fully adjusted model, incorporating all prespecified covariates.

A generalized additive model (GAM) was employed to explore the potential nonlinear relationship between SHR and ICU admission. To assess the robustness of this association across different patient subgroups, interaction and stratified analyses were conducted based on the following baseline characteristics: age, sex, American Society of Anesthesiologists (ASA) physical status classification, body mass index (BMI), and history of hypertension or diabetes mellitus.

To further evaluate the robustness of the association between SHR and ICU admission across different clinical populations, patients were stratified by emergency status (emergency surgery vs. non-emergency surgery) and tumor location (upper gastrointestinal vs. lower gastrointestinal). Within each stratum, multivariable logistic regression was performed to evaluate the association between SHR (per 0.1 unit increase) and ICU admission, adjusting for the same covariates as in Model III. For emergency surgery patients, due to the limited sample size, only crude analysis was performed without multivariable adjustment to avoid model overfitting.

To evaluate the predictive performance of the multivariable model, we constructed receiver operating characteristic (ROC) curves and calculated the area under the curve (AUC) with 95% confidence intervals. Additionally, calibration curves were generated using bootstrapping with 500 resamples to evaluate the agreement between predicted probabilities and observed outcomes. The mean absolute error was calculated to quantify the calibration performance.

To assess the robustness of our findings, we performed several sensitivity analyses. First, a complete case analysis was conducted including only patients with complete data for all covariates. Second, we repeated the analysis after excluding estimated blood loss from the model to evaluate whether the results were driven by this variable with a relatively high missing rate (21.9%). Third, we repeated the analysis after excluding diabetic patients to assess whether the association between SHR and ICU admission was influenced by pre-existing diabetes. All sensitivity analyses used the same multivariable model as the primary analysis (Model 3), with SHR modeled as a continuous variable (per 0.1 unit increase).

All statistical analyses were performed using R software (version 4.4.2) and Free Statistics software (version 2.3) (46). A two-sided *p* value <0.05 was considered statistically significant.

## 3 Results

### 3.1 Study subjects and demographic characteristics

**Figure 1:**
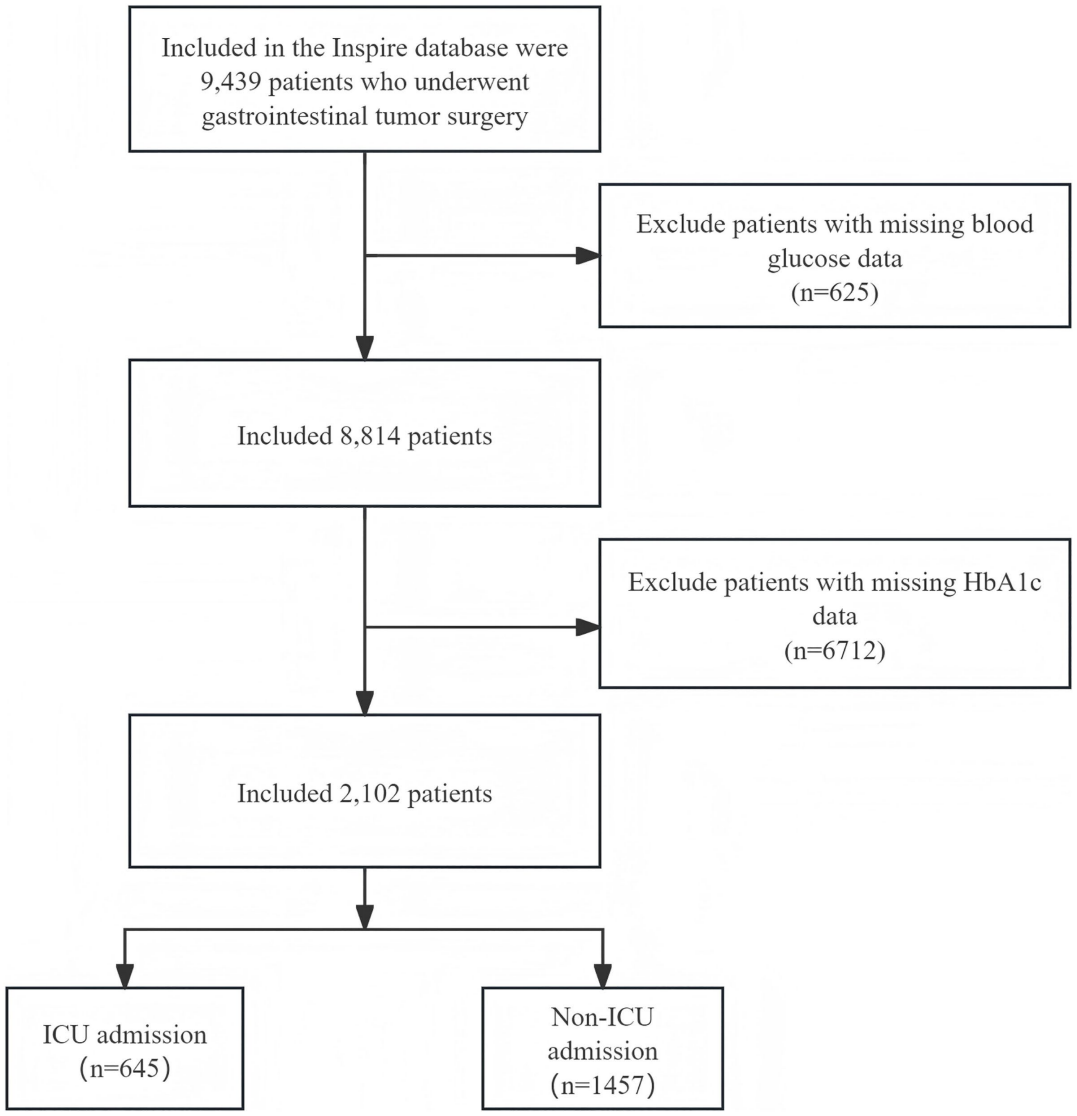
Schematic representation of the participant selection process. Initially, 9,439 patients undergoing gastrointestinal tumor surgery were identified from the INSPIRE database. After excluding patients with missing blood glucose data (*n* = 625) and missing HbA1c data (*n* = 6,712), a total of 2,102 patients were included in the final analytical cohort, comprising 1,457 non-ICU admission patients and 645 ICU admission patients.

This study initially enrolled 9,439 patients (aged 18–90 years) who underwent gastrointestinal tumor surgery. After applying the inclusion and exclusion criteria, a total of 2,102 patients were included in the final analysis (Figure 1).

Table 1 presents the baseline characteristics of the study cohort stratified by tertiles of the SHR. The final analytical cohort comprised 2,102 patients, with a mean age of 66.8 ± 9.6 years; 68.7% were male. Compared to patients in the lower SHR tertiles, those in the highest tertile exhibited a higher American Society of Anesthesiologists (ASA) physical status classification, were more likely to undergo emergency surgery, and demonstrated distinct laboratory profiles characterized by elevated white blood cell count, lower levels of total protein and hemoglobin, and more pronounced electrolyte imbalances, primarily manifested as abnormalities in serum potassium, sodium, and calcium levels. Consequently, this group had a significantly higher incidence of postoperative ICU admission.

**Table 1:** Baseline characteristics of participants.

Variables	Total (*n* = 2,102)	SHR<0.84 (*n* = 694)	0.84 ≤ SHR<1.04 (*n* = 703)	SHR≥1.04 (*n* = 705)	*P* value
Age (years)	66.8 ± 9.6	66.5 ± 9.7	66.4 ± 9.9	67.5 ± 9.2	0.064
Sex, *n* (%)					0.658
Female	658 (31.3)	226 (32.6)	218 (31)	214 (30.4)	
Male	1,444 (68.7)	468 (67.4)	485 (69)	491 (69.6)	
BMI (kg/m^2^)	24.9 ± 53.6	28.4 ± 93.1	23.5 ± 3.8	22.9 ± 4.1	0.105
ASA classification, *n* (%)					<0.001
I	256 (12.2)	93 (13.4)	108 (15.4)	55 (7.8)	
II	1,461 (69.5)	466 (67.1)	485 (69)	510 (72.3)	
III–IV	385 (18.3)	135 (19.5)	110 (15.6)	140 (19.9)	
General anesthesia, *n* (%)					0.36
No	1929 (91.8)	645 (92.9)	639 (90.9)	645 (91.5)	
Yes	173 (8.2)	49 (7.1)	64 (9.1)	60 (8.5)	
Operation time (min)	180.4 ± 129.2	181.3 ± 124.7	175.6 ± 128.9	184.3 ± 133.8	0.433
EmOP, *n* (%)					0.004
No	1952 (92.9)	654 (94.2)	662 (94.2)	636 (90.2)	
Yes	150 (7.1)	40 (5.8)	41 (5.8)	69 (9.8)	
Vasoactive drugs (intraop.), *n* (%)					0.114
No	2042 (97.1)	675 (97.3)	689 (98)	678 (96.2)	
Yes	60 (2.9)	19 (2.7)	14 (2)	27 (3.8)	
Heart rate (bpm)	78.3 ± 14.5	77.9 ± 14.2	78.5 ± 14.6	78.6 ± 14.9	0.638
SBP (mmHg)	127.1 ± 18.7	128.2 ± 18.6	125.5 ± 18.8	127.6 ± 18.7	0.017
Resp (bpm)	17.8 ± 1.4	17.9 ± 1.7	17.7 ± 1.2	17.8 ± 1.3	0.001
Spo_2_ (%)	97.3 ± 2.0	97.3 ± 1.9	97.4 ± 1.9	97.1 ± 2.0	0.004
Hb (g/dL)	12.0 ± 1.9	11.9 ± 1.9	12.2 ± 1.8	11.9 ± 2.0	<0.001
WBC (×10^9/L)	7.8 ± 3.4	7.7 ± 3.3	7.5 ± 3.2	8.1 ± 3.6	0.006
TP (g/dL)	6.5 ± 0.8	6.5 ± 0.8	6.6 ± 0.8	6.4 ± 0.8	<0.001
TBIL (μmol/L)	0.8 ± 0.6	0.7 ± 0.5	0.8 ± 0.7	0.8 ± 0.7	0.026
Scr (mg/dL)	1.1 ± 0.8	1.1 ± 0.9	1.1 ± 0.8	1.1 ± 0.8	0.666
K (mmol/L)	4.2 ± 0.5	4.2 ± 0.4	4.2 ± 0.5	4.2 ± 0.5	0.027
Na (mmol/L)	138.5 ± 3.2	138.9 ± 2.9	138.7 ± 3.0	138.0 ± 3.5	<0.001
Ca (mmol/L)	8.8 ± 0.6	8.8 ± 0.6	8.8 ± 0.6	8.7 ± 0.6	<0.001
APTT (s)	30.9 ± 5.4	30.6 ± 4.1	31.6 ± 6.9	30.4 ± 4.8	<0.001
INR (s)	1.0 ± 0.2	1.0 ± 0.2	1.0 ± 0.2	1.0 ± 0.2	0.451
Hypertension, *n* (%)					0.845
No	1894 (90.1)	629 (90.6)	631 (89.8)	634 (89.9)	
Yes	208 (9.9)	65 (9.4)	72 (10.2)	71 (10.1)	
DM, *n* (%)					0.4
No	1888 (89.8)	621 (89.5)	640 (91)	627 (88.9)	
Yes	214 (10.2)	73 (10.5)	63 (9)	78 (11.1)	
CVD, *n* (%)					0.261
No	2040 (97.1)	668 (96.3)	687 (97.7)	685 (97.2)	
Yes	62 (2.9)	26 (3.7)	16 (2.3)	20 (2.8)	
COPD, *n* (%)					0.029
No	2046 (97.3)	680 (98)	675 (96)	691 (98)	
Yes	56 (2.7)	14 (2)	28 (4)	14 (2)	
CKD, *n* (%)					0.971
No	2061 (98.0)	680 (98)	690 (98.2)	691 (98)	
Yes	41 (2.0)	14 (2)	13 (1.8)	14 (2)	
EBL, mL	100.0 (40.0, 150.0)	100.0 (40.0, 150.0)	100.0 (40.0, 150.0)	100.0 (40.0, 150.0)	0.891
Infusion volume, mL	400.0 (300.0, 550.0)	400.0 (300.0, 550.0)	400.0 (300.0, 550.0)	400.0 (300.0, 550.0)	0.328
ICU admission, *n* (%)					0.001
No	1,457 (69.3)	486 (70)	516 (73.4)	455 (64.5)	
Yes	645 (30.7)	208 (30)	187 (26.6)	250 (35.5)	

Continuous variables are presented as mean ± SD or median (interquartile range), and categorical variables as number (%). Between-group comparisons were performed using ANOVA, Kruskal-Wallis test, chi-square test, or Fisher’s exact test, as appropriate. A *P* value <0.05 was considered statistically significant.

BMI, body mass index; ASA classification, American Society of Anesthesiologists classification; EmOP, emergency operation; Vasoactive drugs (intraop.), intraoperative use of vasoactive drugs; SBP, systolic blood pressure; Resp., respiratory; Spo_2_, pulse oximetry derived oxygen saturation; Hb, hemoglobin; WBC, white blood cell; TP, total protein; TBIL, total bilirubin; Scr, serum creatinine; K, potassium; Na, sodium; Ca, calcium; APTT, activated partial thromboplastin time; INR, international normalized ratio; DM, diabetes mellitus; CVD, cardiovascular disease; COPD, chronic obstructive pulmonary disease; CKD, chronic kidney disease; EBL, estimated blood loss; ICU admission, Intensive Care Unit admission.

### 3.2 Logistic regression analysis of the risk of ICU admission after surgery

The univariable logistic regression results are presented in Supplementary Table S2, identifying multiple significant predictors (*p* < 0.05) of ICU admission, including age, sex, BMI, ASA class III-IV, general anesthesia, operation time, emergency surgery (EmOP), systolic blood pressure (SBP), respiratory rate, SpO₂, hemoglobin (Hb), white blood cell count (WBC), total protein (TP), serum creatinine (Scr), potassium (K), sodium (Na), calcium (Ca), international normalized ratio (INR), and SHR.

**Figure 2:**
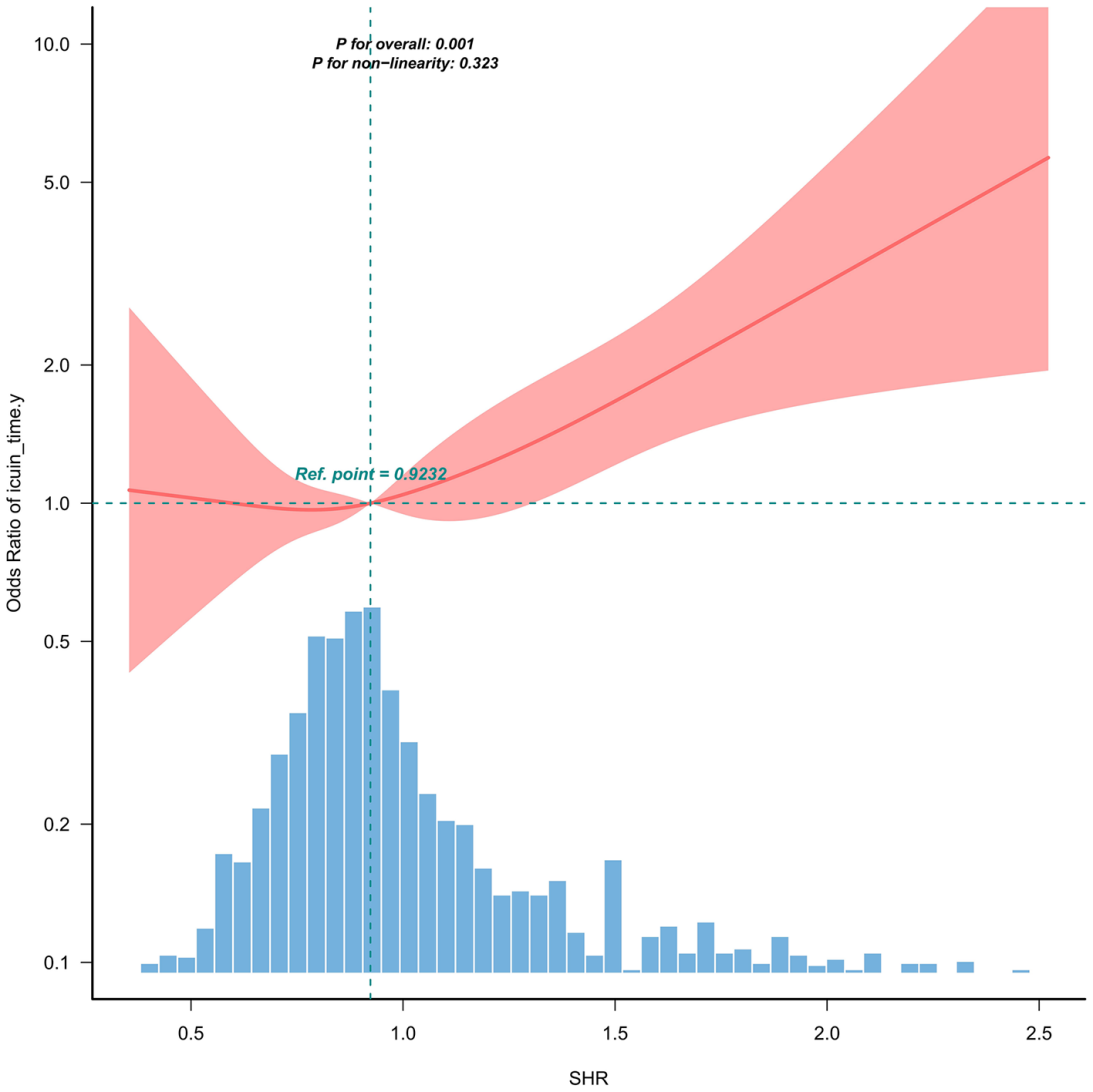
Linear dose–response relationship between SHR and ICU admission. Restricted cubic splines were used to model the dose–response relationship. Adjustments were made for age, sex, BMI, ASA classification, general anesthesia, operation time, EmOP, vasoactive drugs (intraop.), heart rate, SBP, Resp., Spo_2_, Hb, WBC, TP, TBIL, Scr, K, Na, Ca, APTT, INR, hypertension, DM, CVD, COPD, CKD, EBL, infusion volume. The *y*-axis represents the adjusted odds ratio on a logarithmic scale. The red line and red area represent the estimated values and their corresponding 95% confidence intervals, respectively. The histogram at the bottom shows the frequency distribution of SHR. *P* for overall association = 0.001, *P* for non-linearity = 0.323, indicating a linear relationship.

As detailed in Table 2, SHR remained a strong and independent predictor of postoperative ICU admission in multivariable analyses. When modeled as a continuous variable, each 0.1-unit increase in SHR was associated with an adjusted odds ratio (OR) of 1.08 (95% CI: 1.04–1.13, *p* < 0.001) for ICU admission. Similarly, when analyzed categorically, patients in the highest SHR tertile (≥1.04) had an OR of 1.43 (95% CI: 1.04–1.97, *p* = 0.03) compared to those in the lowest tertile. Furthermore, a restricted cubic spline analysis revealed a linear dose–response relationship between SHR levels and ICU admission, as the test for nonlinearity was not significant (P for nonlinearity > 0.05), as illustrated in Figure 2.

**Table 2:** The association between SHR and ICU admission in different models.

Variable	Crude model (*n* = 2,102)		Model I (*n* = 2,102)		Model II (*n* = 2,102)		Model III (*n* = 2,102)	
	OR (95% CI)	*P-*value	OR (95% CI)	*P-*value	OR (95% CI)	*P-*value	OR (95% CI)	*P-*value
SHR (continuous)	1.04 (1.01–1.07)	0.004	1.08 (1.04–1.12)	<0.001	1.08 (1.04–1.13)	<0.001	1.08 (1.04–1.13)	<0.001
Tertiles								
SHR < 0.84	Ref		Ref		Ref		Ref	
0.84 ≤ SHR < 1.04	0.85 (0.67 ~ 1.07)	0.162	0.91 (0.66 ~ 1.25)	0.564	0.88 (0.64 ~ 1.23)	0.460	0.88 (0.63 ~ 1.23)	0.455
SHR ≥ 1.04	1.28 (1.03 ~ 1.61)	0.029	1.36 (1 ~ 1.85)	0.053	1.41 (1.02 ~ 1.94)	0.036	1.43 (1.04 ~ 1.97)	0.030
P for trend		0.025		0.049		0.035		0.030

Data are presented as odds ratios (OR) with 95% confidence intervals (CI) from logistic regression models. *P*-values are reported to three decimal places.

Crude model was not adjusted.

Model 1 was adjusted for age + ASA classification + Operation time + Spo_2_ + TP + Na.

Model 2 was adjusted for model 1 + Sex + BMI + General anesthesia + EmOP + Heart rate + SBP + Resp + Hb + WBC + Scr + K + Ca + INR + EBL.

Model 3 was adjusted for model 2 + Vasoactive drugs (intraop.) + TBIL + APTT + Hypertension + DM + CVD + COPD + CKD + Infusion volume.

BMI, body mass index; ASA classification, American Society of Anesthesiologists classification; EmOP, emergency operation; Vasoactive drugs (intraop.), intraoperative use of vasoactive drugs; SBP, systolic blood pressure; Resp., respiratory; Spo_2_, pulse oximetry derived oxygen saturation; Hb, hemoglobin; WBC, white blood cell; TP, total protein; TBIL, total bilirubin; Scr, serum creatinine; K, potassium; Na, sodium; Ca, calcium; APTT, activated partial thromboplastin time; INR, international normalized ratio; DM, diabetes mellitus; CVD, cardiovascular disease; COPD, chronic obstructive pulmonary disease; CKD, chronic kidney disease; EBL, estimated blood loss.

### 3.3 Subgroup analysis and interactions

**Figure 3:**
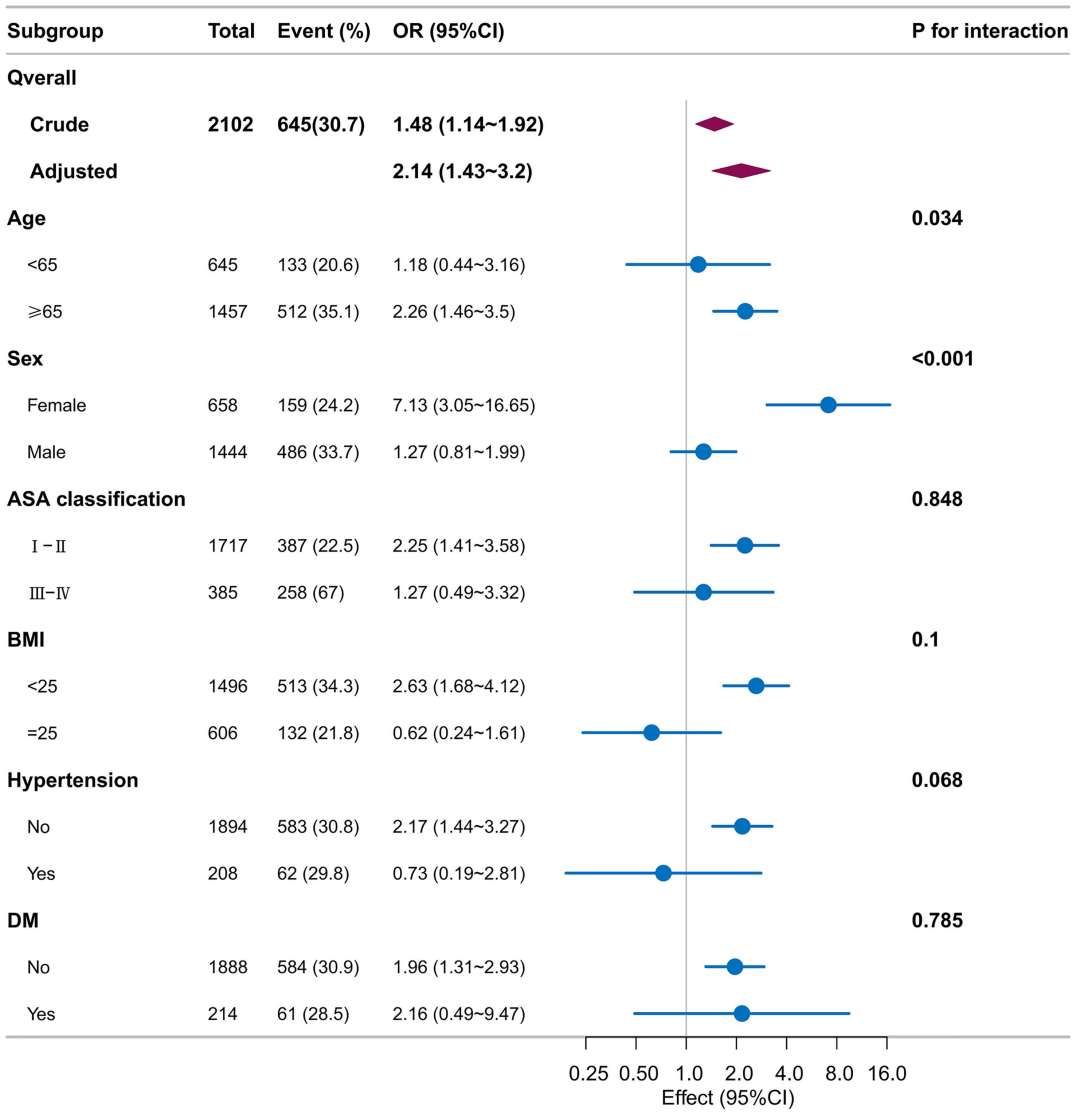
Subgroup analysis of the association between SHR and ICU admission. Forest plot showing adjusted ORs (95% CIs) for SHR across subgroups. Diamond size reflects the weight (sample size) of each subgroup; horizontal lines represent 95% CIs. Interaction *p*-values test for heterogeneity across subgroups. ASA, American Society of Anesthesiologists; BMI, body mass index; DM, diabetes mellitus; OR, odds ratio; CI, confidence interval. Key findings: Significant interactions were observed for age (*p* = 0.034) and sex (*p* = 0.042). No other significant interactions were found. Biases: Estimates from smaller subgroups are less precise and should be interpreted cautiously.

Stratified and interaction analyses were performed to examine the consistency of the association between SHR and postoperative ICU admission across key patient subgroups. These subgroups were defined by age (<65 vs. ≥65 years), sex (male vs. female), ASA physical status classification (I/II vs. ≥III), body mass index (<25 vs. ≥25 kg/m^2^), and histories of hypertension and diabetes. Interaction testing revealed statistically significant interaction effects for age (P for interaction = 0.034) and sex (P for interaction < 0.001). The specific OR and 95% confidence intervals for each subgroup are presented in Figure 3: age <65 years (OR = 1.18, 95% CI: 0.44–3.36), age ≥65 years (OR = 2.26, 95% CI: 1.44–3.50); male (OR = 1.27, 95% CI: 0.81–1.99), female (OR = 7.13, 95% CI: 3.05–16.65). No other significant interaction effects were detected in the remaining subgroups.

### 3.4 Stratified analysis

In stratified analysis by emergency status (Supplementary Table S3), the independent association between SHR and ICU admission remained significant among non-emergency surgery patients (*n* = 1,952) after full multivariable adjustment (per 0.1 unit increase: OR = 1.08, 95% CI: 1.04–1.13, *p* < 0.001). In the emergency surgery stratum (*n* = 150), due to limited sample size, multivariable adjustment was not performed; crude analysis showed no significant association (OR = 1.11, 95% CI: 0.56–2.21, *p* = 0.764).

Stratified by tumor location (Supplementary Table S3), the association between SHR and ICU admission remained significant in both upper gastrointestinal tumor patients (OR = 1.08, 95% CI: 1.02–1.14, *p* < 0.001) and lower gastrointestinal tumor patients (OR = 1.08, 95% CI: 1.03–1.14, *p* < 0.001).

### 3.5 Predictive performance of the multivariable model

**Figure 4:**
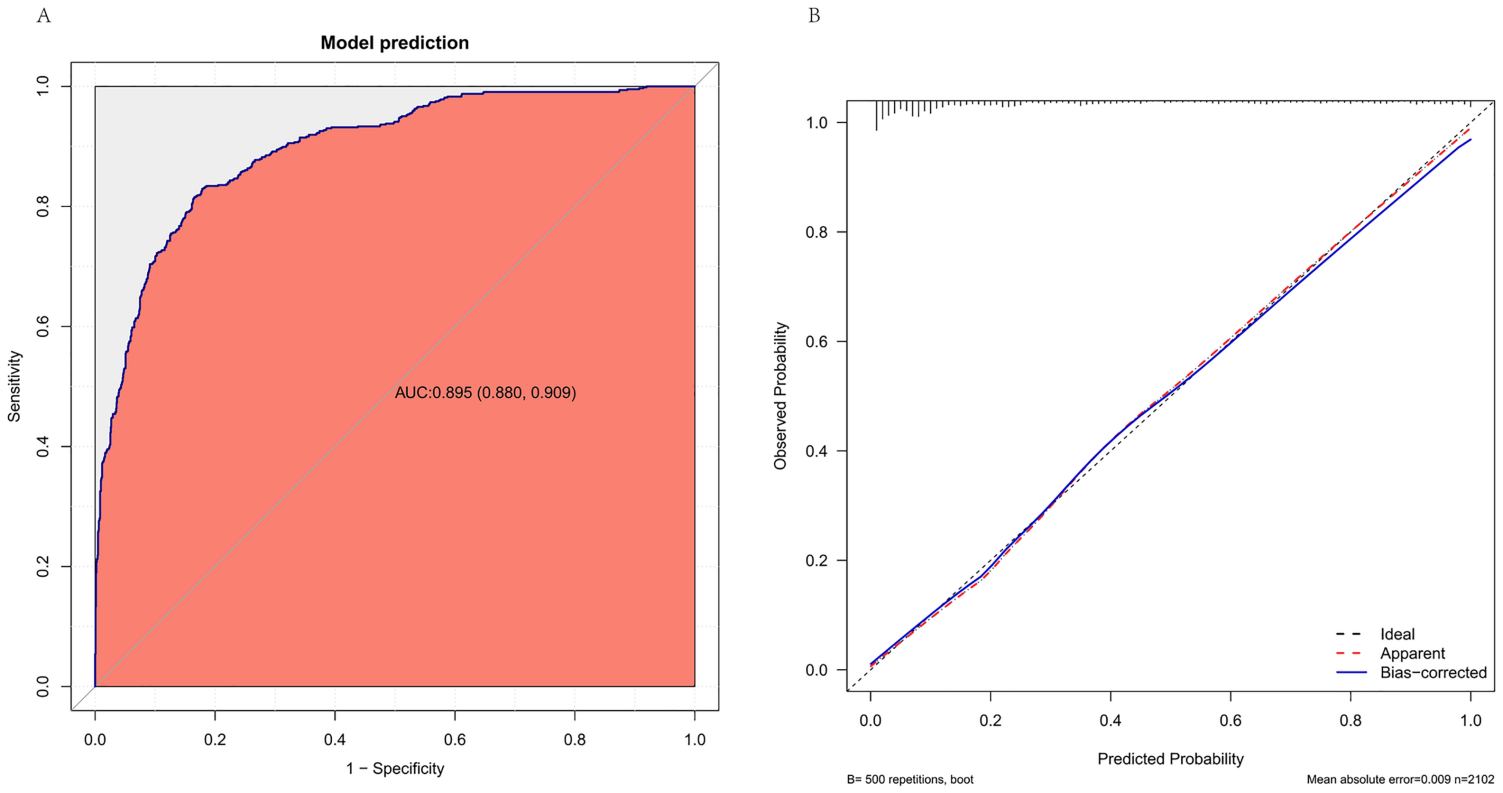
Performance of the prediction model for postoperative ICU admission. **(A)** ROC curve demonstrating the discrimination of the model. The AUC was 0.895 (95% CI: 0.88–0.91). **(B)** Calibration plot with 500 bootstrap resamples. The bias-corrected curve is close to the ideal diagonal, indicating good calibration. Mean absolute error = 0.009.

The predictive performance of the model was evaluated using ROC curve and calibration curve (Figure 4). The AUC was 0.895 (95% CI: 0.880–0.909), indicating excellent discrimination (Figure 4A). The calibration plot demonstrated good agreement between predicted and observed outcomes, with a mean absolute error of 0.009 (Figure 4B).

### 3.6 Sensitivity analysis

Sensitivity analyses yielded consistent results with the primary analysis (Supplementary Table S4). Complete case analysis (*n* = 1,504) showed that each 0.1 unit increase in SHR was significantly associated with increased odds of ICU admission (OR = 1.10, 95% CI: 1.05–1.16, *p* < 0.001). After excluding estimated blood loss from the model (*n* = 1,887), the effect of SHR remained significant (OR = 1.08, 95% CI: 1.04–1.13, *p* < 0.001). After excluding diabetic patients (*n* = 1,888), the association also persisted (OR = 1.10, 95% CI: 1.05–1.16, *p* < 0.001). These findings demonstrate that the primary results are robust and not dependent on the method of handling missing data or the inclusion of specific variables.

## 4 Discussion

This real-world cohort study demonstrates that an elevated preoperative SHR is independently associated with postoperative ICU admission in patients undergoing surgery for gastrointestinal tumors. Elevated SHR was consistently associated with ICU transfer, both when analyzed as a continuous and as a categorical variable. Restricted cubic spline analysis supported a linear dose–response relationship between SHR levels and ICU admission. The SHR-incorporated model demonstrated excellent discrimination and calibration, supporting its utility for accurate risk stratification and clinical decision-making.

Stress hyperglycemia is a transient metabolic response elicited by neuroendocrine activation following acute physiological insults such as trauma or major surgery (24, 25). The core pathophysiology involves surgery-induced insulin resistance and a surge in counterregulatory hormones, including cortisol and catecholamines (26–28). Beyond directly elevating blood glucose, these perturbations impair tissue repair and compromise immune competence through multiple interconnected mechanisms. These encompass monocyte dysfunction, mitochondrial impairment, enhanced release of pro-inflammatory cytokines (e.g., interleukin-6), and aggravated oxidative stress (29, 30).

Although an initial hyperglycemic response may provide rapid energy supply and confer transient physiological benefits, sustained hyperglycemia is associated with various adverse clinical outcomes, most notably the exacerbation of systemic pro-inflammatory responses (31, 32). Conventional single-timepoint blood glucose measurements, whether obtained at admission or preoperatively, are often inadequate for capturing the dynamic fluctuations and true magnitude of glycemic stress. Therefore, the SHR, as an indicator that corrects for baseline blood glucose levels, provides a crucial basis for a more objective assessment of the severity of stress-induced hyperglycemia (12).

Our finding that elevated SHR is associated with postoperative ICU admission in gastrointestinal tumor surgery patients is consistent with its established prognostic value across diverse clinical contexts. For instance, a meta-analysis of hospitalized COVID-19 patients reported that a high SHR was associated with a nearly two-fold increased risk of ICU admission (33). In perioperative medicine, SHR has shown a U-shaped association with 30- and 90-day major adverse cardiac and cerebrovascular events after non-cardiac surgery, where both excessively high and low values signify elevated risk (10). Similarly, among orthopedic surgery patients, elevated SHR correlates with adverse outcomes including cardiovascular events, systemic infections, and prolonged hospitalization, irrespective of diabetic status (11). The utility of SHR extends to other specialties. In cardiology, it is associated with major adverse cardiovascular events following percutaneous coronary intervention (PCI) (34) and predicts new-onset atrial fibrillation after coronary artery bypass grafting (CABG) (35). Furthermore, SHR is linked to both short- and long-term outcomes in heart failure (36, 37). In neurology, higher SHR levels are correlated with poor functional recovery, hemorrhagic transformation, and stroke-associated pneumonia in patients with acute ischemic stroke (38–40). Beyond these, SHR has also been identified as a risk factor for clinical deterioration in chronic thromboembolic pulmonary hypertension (41) and for incident delirium in elderly hospitalized patients (42).

This study found significant interaction effects of age and sex on the association between SHR and ICU admission, with the predictive effect being significant only in elderly and female patients. The age interaction may stem from diminished physiological reserve and impaired metabolic regulation in elderly patients. With advancing age, pancreatic *β*-cell function declines and insulin resistance worsens (43), limiting compensatory capacity under stress, allowing SHR to better reflect a state of decompensation. In contrast, younger patients possess stronger regulatory capacity, and fluctuations in SHR may not necessarily translate into clinical risk. Regarding the sex interaction, the OR for females was as high as 7.13, far exceeding that for males. This may be related to the decline in estrogen levels after menopause, which leads to the loss of its protective effects on insulin sensitivity and vascular function (44). The female patients in this study were predominantly postmenopausal, and their baseline insulin resistance may be more pronounced than in premenopausal women, potentially amplifying the predictive value of SHR under surgical stress. However, the wide confidence interval in the female subgroup suggests the need for further validation. The predictive value of SHR appears to be population-specific. Clinical application should consider age and sex differences, and future studies with larger sample sizes are needed for validation.

Of note, the association between SHR and ICU admission remained consistent in patients with upper and lower gastrointestinal tumors. Upper gastrointestinal surgery typically involves extensive manipulation within the thoracic or abdominal cavity, resulting in greater surgical trauma and a higher risk of postoperative complications such as anastomotic leakage and respiratory insufficiency. Consequently, ICU admission in these patients may partially reflect a prophylactic monitoring requirement for high-risk procedures. In contrast, lower gastrointestinal surgery is associated with relatively less trauma, and postoperative ICU admission in this group is more often for monitoring potential complications rather than for mandatory life support (45). The finding that SHR significantly predicted ICU admission in both upper and lower gastrointestinal tumor patients in this study suggests that it captures the patient’s intrinsic stress response capacity, rather than merely reflecting routine monitoring needs dictated by the surgical site.

In summary, the SHR has demonstrated broad clinical utility across diverse specialties—spanning perioperative care, critical care, cardiology, and neurology—with accumulating evidence supporting its role as a promising cross-disciplinary prognostic marker. The present study extends this applicability to the specific domain of gastrointestinal cancer surgery, suggesting that preoperative SHR assessment may serve as a useful indicator for stratifying postoperative critical care risk.

## 5 Conclusion

The preoperative SHR is independently associated with postoperative transfer to the intensive care unit among patients undergoing gastrointestinal tumor surgery. Notably, 30.7% (645/2,102) of patients in our cohort required ICU admission following surgery, underscoring the clinical importance of preoperative risk stratification. The multivariable model incorporating SHR demonstrated good predictive performance, suggesting its potential clinical utility for risk stratification. Incorporating SHR into preoperative assessment facilitates early identification of high-risk patients and optimizes perioperative management.

## 6 Limitations

Several limitations of this study merit consideration. First, the data originate from a single-center registry, which may limit the generalizability of our findings to other populations with different healthcare systems and ICU admission practices. ICU resource allocation and admission criteria vary considerably across countries, and our results should be validated in diverse international settings. Second, the study design itself may have introduced multiple biases. The application of inclusion and exclusion criteria could lead to selection bias. In addition, despite comprehensive adjustment for known confounders, the retrospective design cannot rule out residual confounding from unmeasured variables or information bias, particularly regarding the accuracy and completeness of recorded clinical variables. Third, several clinically important factors that may influence SHR levels were not included in this study, including tumor type and stage, whether preoperative therapy was received, and the type of diabetes treatment. These factors may affect the degree of stress hyperglycemia: upper gastrointestinal malignancies may alter baseline glycemic status by affecting food intake, and different types of glucose-lowering therapy may also influence the interpretation of SHR. Future studies should collect this information to more comprehensively evaluate the independent predictive value of SHR. Fourth, the primary outcome was postoperative ICU admission as a composite endpoint, without differentiation based on specific clinical indications. In particular, this study was unable to directly distinguish between planned and unplanned ICU admissions, which is an important limitation when interpreting the clinical utility of SHR. Future studies should investigate the association between SHR and distinct etiologies of postoperative deterioration, including the collection of detailed data on ICU admission indications, to elucidate potential pathophysiological mechanisms and further validate our findings.

## Data Availability

Publicly available datasets were analyzed in this study. This data can be found here: https://physionet.org/content/inspire/1.3/.
