# Supplementary material for: Elevated stress hyperglycemia ratio predicts intensive care unit admission after surgery for gastrointestinal tumors: an INSPIRE database analysis
Source: Front Med (Lausanne). 2026 Mar 16;13:1794194. doi: 10.3389/fmed.2026.1794194 (PMC13033595; doi:10.3389/fmed.2026.1794194)
Supplement: Supplementary file 1 [file Table_1.docx]

**TABLE S1** | Variance inflation factor of each variable in the entire cohort.

| **Variables** | **Variance inflation factor (VIF)** |
| --- | --- |
| **Age** | 1.177 |
| **Sex** | 1.201 |
| **BMI** | 1.186 |
| **ASA classification** | 1.121 |
| **General anesthesia** | 1.313 |
| **Operation time** | 1.163 |
| **EmOP** | 1.281 |
| **Vasoactive drugs (intraop.)** | 1.22 |
| **Heart rate** | 1.024 |
| **SBP** | 1.13 |
| **Resp** | 1.132 |
| **Spo_2_** | 1.073 |
| **Hb** | 1.181 |
| **WBC** | 1.586 |
| **TP** | 1.315 |
| **TBIL** | 2.036 |
| **Scr** | 1.14 |
| **K** | 1.354 |
| **Na** | 1.203 |
| **Ca** | 1.296 |
| **APTT** | 2.005 |
| **INR** | 1.446 |
| **Hypertension** | 1.496 |
| **DM** | 1.149 |
| **CVD** | 1.096 |
| **COPD** | 1.115 |
| **CKD** | 1.032 |
| **EBL** | 1.087 |
| **Infusion volume** | 1.597 |

**Abbreviations:** BMI, body mass index; ASA **classification**, american society of anesthesiologists **classification; EmOP, emergency operation; Vasoactive drugs (intraop.), intraoperative use of vasoactive drugs;** SBP, systolic blood pressure; Resp, respiratory; Spo_2_, pulse oximetry derived oxygen saturation; Hb, hemoglobin; WBC, white blood cell;TP, t**otal protein;** TBIL, total bilirubin; Scr, serum creatinine; K, potassium; Na, sodium; Ca, calcium; APTT, activated partial thromboplastin time; INR, international normalized ratio; DM, diabetes mellitus; CVD, cardiovascular disease; COPD, chronic obstructive pulmonary disease; CKD, c**hronic kidney diseas**e, EBL, estimated blood loss.

**TABLE S2** | **Univariate analysis of ICU admission in patients undergoing gastrointestinal tumor surgery.**

| **Variables** | **OR(95%CI)** | ***P-*value** |
| --- | --- | --- |
| **Age** | 1.05 (1.04~1.06) | <0.001 |
| **Sex** | 1.59 (1.29~1.96) | <0.001 |
| **BMI** | 0.95 (0.92~0.97) | <0.001 |
| **ASA classification** |  |  |
| **Ⅰ** | Ref |  |
| **Ⅱ** | 1.07 (0.78~1.48) | 0.661 |
| **Ⅲ-Ⅳ** | 7.42 (5.15~10.71) | <0.001 |
| **General anesthesia** | 0.04 (0.01~0.11) | <0.001 |
| **Operation time** | 1.01 (1.01~1.01) | <0.001 |
| **EmOP** | 1.47 (1.04~2.06) | 0.029 |
| **Vasoactive drugs (intraop.)** | 0.62 (0.33~1.15) | 0.128 |
| **Heart rate** | 1.01 (1~1.01) | 0.096 |
| **SBP** | 0.99 (0.99~1) | 0.001 |
| **Resp** | 1.13 (1.06~1.2) | <0.001 |
| **Spo_2_** | 0.88 (0.84~0.92) | <0.001 |
| **Hb** | 0.87 (0.83~0.92) | <0.001 |
| **WBC** | 0.97 (0.94~0.99) | 0.016 |
| **TP** | 0.73 (0.65~0.81) | <0.001 |
| **TBIL** | 1.1 (0.96~1.27) | 0.16 |
| **Scr** | 1.3 (1.17~1.44) | <0.001 |
| **K** | 1.33 (1.09~1.63) | 0.005 |
| **Na** | 0.93 (0.9~0.96) | <0.001 |
| **Ca** | 0.71 (0.61~0.83) | <0.001 |
| **APTT** | 1.01 (0.99~1.03) | 0.197 |
| **INR** | 9.98 (5.53~17.98) | <0.001 |
| **Hypertension** | 0.95 (0.7~1.31) | 0.773 |
| **DM** | 0.89 (0.65~1.22) | 0.466 |
| **CVD** | 1 (0.58~1.73) | 0.994 |
| **COPD** | 0.9 (0.5~1.62) | 0.728 |
| **CKD** | 1.31 (0.69~2.49) | 0.409 |
| **EBL (per 100 mL increment)** | 0.91 (0.82~1.01) | 0.065 |
| **Infusion volume (per 100 mL increment)** | 1.01 (0.98~1.04) | 0.457 |
| **SHR** | 1.48 (1.14~1.92) | 0.004 |

**Abbreviations:** BMI, body mass index; ASA **classification**, american society of anesthesiologists **classification; EmOP, emergency operation; Vasoactive drugs (intraop.), intraoperative use of vasoactive drugs;** SBP, systolic blood pressure; Resp, respiratory; Spo_2_, pulse oximetry derived oxygen saturation; Hb, hemoglobin; WBC, white blood cell;TP, t**otal protein;** TBIL, total bilirubin; Scr, serum creatinine; K, potassium; Na, sodium; Ca, calcium; APTT, activated partial thromboplastin time; INR, international normalized ratio; DM, diabetes mellitus; CVD, cardiovascular disease; COPD, chronic obstructive pulmonary disease; CKD, c**hronic kidney disease;** EBL, estimated blood loss; **SHR, stress hyperglycemia ratio.**

**TABLE S3** | **Subgroup-specific multivariable analysis of the association between SHR and ICU admission**

| **Subgroup** | **No. of patients** | **OR (95% CI)*** | **P value** | **Note** |
| --- | --- | --- | --- | --- |
| **Emergency status** |  |  |  |  |
| Emergency surgery | 150 | 1.11 (0.56~2.21) | 0.764 | Crude OR |
| Non-emergency surgery | 1952 | 1.08 (1.04–1.13) | <0.001 | Adjusted OR |
| **Tumor location** |  |  |  |  |
| Upper **Gastrointestinal** | 1008 | 1.08 (1.02–1.14) | 0.009 | Adjusted OR |
| Lower **Gastrointestinal** | 1094 | 1.08 (1.03–1.14) | 0.005 | Adjusted OR |

*OR per 0.1 unit increase in SHR, adjusted for all covariates in Model 3. For emergency surgery patients, only crude OR is reported due to limited sample size to avoid model overfitting.

**TABLE S4 | Sensitivity analysis results**

| Sensitivity analysis | n | OR (95% CI)* | P value |
| --- | --- | --- | --- |
| Primary analysis (multiple imputation) | 2,102 | 1.08(1.04~1.13) | <0.001 |
| Complete case analysis | **1504** | **1.10 (1.05-1.16)** | <0.001 |
| Excluding estimated blood loss | 1887 | **1.08 (1.04-1.13)** | <0.001 |
| Excluding diabetic patients | 1,888 | 1.10 (1.05-1.16) | <0.001 |

*OR per 0.1 unit increase in SHR, adjusted for all covariates in Model 3.
